# Scar Ectopic Pregnancy - An Emerging Challenge

**DOI:** 10.7759/cureus.16673

**Published:** 2021-07-27

**Authors:** Ipsita Mohapatra, Subha R Samantray

**Affiliations:** 1 Obstetrics and Gynecology, All India Institute of Medical Sciences Kalyani, Kalyani, IND

**Keywords:** scar ectopic pregnancy, uterine scar, methotrexate, serum β-hcg, expectant management, laparotomy, hysterectomy

## Abstract

Cesarean scar ectopic pregnancy (SEP), a rare type of ectopic pregnancy, is the implantation of a gestational sac in the myometrium and fibrous tissues at the site of a previous uterine scar (mostly cesarean section scar). The condition can be catastrophic if not managed on time, leading to significant morbidity and mortality. Early diagnosis made by transvaginal ultrasonography and a high degree of suspicion for the probability of SEP in previous uterine surgery patients may help in the initiation and success of conservative treatment, prevention of complications, and preservation of fertility. We present here the analysis of 22 cases of SEP managed at our institute between 2013 to 2020.

The mean gestational age at the time of diagnosis was 8.6±2.2 weeks. The majority of the women presented with either pain or bleeding, but few cases (7 cases) were asymptomatic and were diagnosed with SEP during routine obstetric ultrasonography. Out of these cases, a single case was admitted with shock due to uterine rupture. The mean serum β-hCG level was 29,543 mIU/ml (range, 2105-61590). Asymptomatic patients with low serum β-hCG levels(<15,000 mIU/ml) were given a single dose of methotrexate. Methotrexate was given as a single dose or 4 doses regimen in total 13 cases. Laparotomy with wedge resection of the scar ectopic was done in 8 cases.

Overall primary treatment success was recorded in 20 of 22 cases (91%). 2 cases underwent dilatation and curettage due to retained product of conception after primary treatment with methotrexate. The serum β-hCG levels were normalized in an average time period of 53 days.

This retrospective case series has proved the role of early and accurate diagnosis of SEP for initiating the treatment in order to minimize maternal morbidity and mortality related to this rare and unusual form of ectopic pregnancy.

## Introduction

Cesarean scar ectopic pregnancy, a rare type of ectopic pregnancy, is the implantation of a gestational sac in the myometrium and fibrous tissues at the site of a previous uterine scar (mostly cesarean section scar) [1]. The incidence of scar ectopic pregnancies (SEP) is about 0.15% in women with previous cesarean section, and it accounts for about 6.1% of all ectopic pregnancies in women who had at least one previous cesarean section [2]. 

There is an increasing trend in the incidence of SEP in the past few decades. The probable explanation may be the rising trend in a number of procedures that could potentially damage the endometrium like cesarean section, dilatation and evacuation, myomectomy, metroplasty, operative hysteroscopy, and manual removal of placenta. The second reason for the increasing trend may be the better-diagnosing modalities that are able to detect the SEP with a high degree of precision [3]. The condition can be catastrophic if not managed on time, leading to significant morbidity and mortality. There may be associated complications like placenta accreta and uterine rupture if the pregnancy continues to a higher gestational period.

There is no consensus on the best treatment modality for SEP. Various treatment options have been opted over the years according to the gestational age and clinical features at the time of presentation. Treatment modalities include expectant management; methotrexate administration with or without dilation and curettage; excision of the SEP by vaginal, abdominal, or laparoscopic route; hysterectomy; and many others in different combinations.

Early diagnosis made by transvaginal ultrasonography and a high degree of suspicion for the probability of SEP in previous uterine surgery patients may help in the initiation and success of conservative treatment, prevention of complications, and preservation of fertility. We present here the analysis of 22 cases of SEP managed at our institute between 2013 to 2020.

## Materials and methods

This is a retrospective review of 22 cases of pregnant women between 6-15 weeks of gestation with the diagnosis of scar ectopic pregnancy at a tertiary care hospital between the periods of July 2013 to July 2020. The approval for the study was taken from the institutional ethical committee and institutional review board. All the data were retrieved from the medical records of the patients.

Scar ectopic pregnancy can be diagnosed on the basis of some ultrasonographic features [3-5]. They are visualization of an empty uterine cavity and empty endocervical canal, development of the sac in the anterior wall of the isthmic portion, a discontinuity on the anterior wall of the uterus demonstrated on the sagittal plane of the uterus running through the amniotic sac, and detection of the placenta and/or a gestational sac embedded on the hysterotomy scar. In the early gestational weeks, a triangular gestational sac fills the niche of the scar, and at 8 postmenstrual weeks, this shape may become rounded or even oval. Thin or absent myometrium between the bladder and the sac and negative sliding sac sign (gestational sac position does not change with pressure applied by the transvaginal probe) may be present. A high velocity with low impedance peri-trophoblastic vascular flow clearly surrounding the sac is seen in the Doppler examination.

The following eligibility criteria were taken into consideration: 1) the ultrasound images confirmed the diagnosis, and 2) patients were willing to follow up.

Treatment to be given was decided according to the institutional treatment protocol basing upon the type of SEP, the β-hCG level, vascularity of the SEP, gestational age, size of the sac, and clinical presentation of the patient. Successful treatment was defined as complete resolution of the pregnancy sonographically and decrease in the serum β-hCG levels.

Written informed consent for participation in the study was taken from the patients. Patients who could not be contacted or did not give consent for the study were excluded from the study. The patient’s clinical data, treatment modality, and complications, if any, were analyzed. All statistical analyses were performed with SPSS version 21.0 (IBM Inc., Armonk, New York).

## Results

During the total study period, a total of 22 cases of SEP received treatment at our institute. The detailed characteristic of each case and their composite data are depicted in Table 1. The complete demographic profile and clinical presentation with laboratory parameters have been compiled in Table 2. The mean age of the patients was 30.31 (range, 26-34) years. Out of the total 22 patients, 6 (27%) patients had more than one previous surgery. The patients had either previous cesarean section (22 cases), myomectomy (2 cases), or hysterotomy (1 case). The mean gestational age at the time of diagnosis was 8.6±2.2 weeks. Most of the women presented with either pain or bleeding, but few (7 cases) were asymptomatic and were diagnosed with SEP during routine obstetric ultrasonography. Out of these cases, a single case was admitted with shock due to uterine rupture.

**Table 1:** Detailed description of the cases GA - gestational age, CS - cesarean section

S.No	Age (yrs.)	GA (wks.)	Previous surgeries (nos.)	Type of previous surgery	Time since surgery	Symptoms	Gest. sac (mm)	Perisac vascularity	Cardiac activity	S. βhcg (mIU/ml)
1	28	7	1	CS	2	Pain	16	mild	absent	23300
2	32	6.3	1	CS	3	None	13	mild	absent	14900
3	33	7.1	2	CS	3.2	Pain& Bleeding	17	moderate	absent	34000
4	26	9	1	CS	2.7	Pain& Bleeding	40	moderate	present	28430
5	31	16	1	CS	4	Shock	NA	NA	present	58670
6	28	10	1	CS	3.4	Pain& Bleeding	48	high	present	42399
7	34	11.2	3	CS	2.6	Pain& Bleeding	24	moderate	absent	28303
8	33	6	1	CS	3	None	15	mild	absent	15404
9	29	8	2	CS, hysterotomy	2.8	Pain	30	high	present	32003
10	30	8.3	1	CS	4	Pain	20	mild	present	15200
11	30	9	1	CS	4.1	Pain& Bleeding	31	moderate	absent	30,201
12	31	9.2	1	CS	3	Pain& Bleeding	18	mild	absent	24601
13	26	7.4	1	CS	2.5	None	20	mild	absent	19700
14	29	8	2	CS	3	None	35	moderate	present	31,211
15	31	12.2	1	CS	4.1	Bleeding	40	high	present	61590
16	34	7	2	CS	4.6	None	12	mild	absent	12600
17	28	8.1	2	CS, Myomectomy	2	Pain& Bleeding	21	moderate	absent	26502
18	29	9	1	Myomectomy	3.7	Pain& Bleeding	26	high	present	54850
19	33	6.4	1	CS	2.6	None	12	mild	absent	2105
20	32	7.1	1	CS	3	None	14	mild	absent	19300
21	31	8	1	CS	3.1	Pain	22	moderate	absent	30,484
22	29	9.3	1	CS	2.10	Pain& Bleeding	26	high	present	29526

**Table 2:** The compiled data of all the 22 patients GA - Gestational Age SD - Standard Deviation

Characteristic of patient	Value
No. of patients	22
Age in years (Mean±SD)	30.31±2.3
GA in wks at time of diagnosis (Mean±SD)	8.6±2.2
No of prior surgeries	2(1-3)
Time since previous surgery	3.11±0.72
Gestational sac size in mm (Mean±SD)	23.8±9.98
Presence of cardiac activity	9(41%)
hCG level (mIU/ml)	29542(2105-61590)
Abdominal pain at diagnosis	13(59%)
Vaginal bleeding at diagnosis	10(45%)
Asymptomatic	7(32%)
Vascularity around sac: low, moderate, high	9(41%), 7(32%), 5(23%)

On ultrasonographic examination of the SEP cases, the mean gestational sac diameter was found to be 23.8±9.98 mm. Cardiac activity was present in 9 (41%) cases. The mean serum β-hCG level was 29,543 mIU/ml (range, 2105-61590). There was significant vascularity around the implantation site (moderate in 7 cases; severe in 5 cases).

The type of treatment given along with the complications is tabulated in Table 3. The mode of treatment was decided based upon the clinical presentation, serum β-hCG levels, and ultrasonographic picture. Asymptomatic patients with low serum β-hCG levels (<15,000 mIU/ml) were given a single dose of systemic methotrexate. Methotrexate was given as a single dose or 4 dose regimen along with leucovorin rescue in a total of 13 cases. Laparotomy with wedge resection of the scar ectopic was done in 8 cases. One case who was admitted with shock had to undergo a hysterectomy and postoperative blood transfusion. Overall primary treatment success was recorded in 20 of 22 cases (91%). 2 cases underwent dilatation and curettage due to retained product of conception after primary treatment with methotrexate. The serum β-hCG levels were normalized in an average time period of 53 days.

**Table 3:** Type of treatment and complications Mtx^1 ^- Methotrexate single dose, Mtx^4 ^- Methotrexate 4 doses, RPOC - retained product of conception, D&C - dilatation & curettage

S.no	Treatment	Complications	Additional treatment	Serum β-hCG normalization time (d)
1	Mtx^1^	None	None	87
2	Mtx^1^	None	None	66
3	Mtx^4^	None	None	54
4	Mtx^4^	None	None	80
5	Hysterectomy	Bleeding	Blood transfusion	26
6	Wedge Resection	None	None	32
7	Wedge Resection	None	None	64
8	Mtx^1^	None	None	28
9	Wedge Resection	None	None	88
10	Mtx^1^	RPOC	D&C	56
11	Mtx^1^+D&C	None	None	47
12	Wedge Resection	None	None	70
13	Mtx^1^	None	None	21
14	Mtx^4^	None	None	39
15	Wedge Resection	None	None	49
16	Mtx^1^	None	None	55
17	Wedge Resection	None	None	64
18	Wedge Resection	None	None	42
19	Mtx^1^	None	None	68
20	Mtx^1^	RPOC	D&C	24
21	Mtx^1^+D&C	None	None	63
22	Wedge Resection	None	None	41

## Discussion

Ectopic pregnancy (EP) is a life-threatening obstetrics emergency in the early trimester, associated with high morbidity and mortality if not intervened timely [6]. Among all types of ectopic pregnancies, scar ectopic pregnancy is one of the rarest types. The first case of SEP was reported in 1978 in English medical literature [7]. SEP is defined as a gestation completely surrounded by myometrium and fibrous tissue of the previous uterine scar separated from the endometrial cavity and endocervical canal [8]. In this retrospective cohort study, a total of 22 cases of SEP were evaluated.

The incidence of SEP is on the rise. This is parallel with the increasing trend in the rate of cesarean sections worldwide [9]. So, while explaining to the patient about the complications of CS, scar ectopic pregnancy possibility has also to be included as a remote complication. But it has also been observed by studies that multiple cesarean sections may not further increase the incidence of SEP [10].

There is no clear understanding of the mechanism of SEP. It's thought that there is a formation of a microtubular tract in the uterine scar due to poor healing. The implantation occurs in this defect. In SEP, the gestational sac is completely surrounded by myometrial and fibrotic tissue of the previous uterine scar and is completely separated from the endometrial and endocervical cavities.

SEP can be broadly divided into two types. Type I is endogenic variety, caused by the implantation of the gestational sac within the prior scar with progression towards the cervico-isthmic space or the uterine cavity. It may result in a viable pregnancy but has a very high risk of bleeding at the placental site. Type II is the exogenic variety and is caused by deep implantation of the pregnancy within the scar defect with further growth infiltrating into the uterine myometrium followed by uterine serosal surface and may finally end up in uterine rupture and massive hemorrhage. Type II is a more dangerous and life-threatening condition [11]. The SEP may lose its vascular connections as it grows, leading to spontaneous abortion; or may grow with stronger vascularity, ending in a low-lying adherent placenta with or without invasions into the nearby structures.

The clinical symptoms may vary from being asymptomatic to painless or painful vaginal bleeding. In our study, 7 cases (32%) were asymptomatic. Sometimes the condition remains undiagnosed as the patients are asymptomatic, and then they present late with uterine rupture or massive hemorrhage. So, every woman with a history of uterine surgery should be screened early in the first trimester of pregnancy [12]. Transvaginal ultrasonography and color flow Doppler are essential for the early diagnosis of SEP. Color flow Doppler can be used to demonstrate the peri-trophoblastic flow around the gestational sac and its relationship to the uterine scar and nearby viscera [2]. In the present study, the mean gestational age at diagnosis of SEP was 8.6±2.2 weeks. The mean gestational age at diagnosis in a systemic review done by Gonzalez and Tulandi was reported to be 7±2.5 weeks [12]. There is a higher risk of complication rate in case of late diagnosis, probably due to higher serum β-hCG levels and increased vascularity.

The factors which may be associated with massive hemorrhage in SEP are multiple gravidities, the greater maximum diameter of gestation sac, high gestational age, high β -hCG level, and high flow around the lesion. The SEP cases with thick myometrium have less chance of heavy bleeding [13].

There is no defined optimal treatment modality of SEP to date due to limited reports with large numbers and the paucity of well-designed randomized controlled trials. The aim of the treatment of SEP is to prevent complications, conserve the uterus for future fertility, and maintenance of the healthy quality of life of the woman concerned. The treatment option chosen depends on various factors like the gestational age, hemodynamic status, expertise of the treating surgeon, desire for future fertility, willingness for follow up and finally, accepting the chances of complications. A systemic review done by Timor-Tritsch and Monteagudo identified a total of 31 different treatment modalities for SEP [14]. The various treatment options carried out all over the world are expectant management, systemic methotrexate, local methotrexate, dilatation and evacuation, uterine artery embolization, hysteroscopy, laparoscopy, laparotomy with wedge resection, and finally hysterectomy for massive hemorrhage or uterine rupture.

Expectant management is usually not recommended due to the high chances of serious complications as the pregnancy progresses. A systemic review and meta-analysis done by Cali et al. suggested that expectant management may be a reasonable option for cesarean scar pregnancy (CSP) with no detectable embryonic/fetal cardiac activity as 70% of the included cases in the meta-analysis did not experience any major maternal complication and had an uncomplicated abortion. But serial monitoring of serum β-hCG is mandatory for such cases until normalization. They also concluded that CSP with cardiac activity has a high burden of complications if on expectant management [15]. In our study, none of the patients were given expectant management as the patient did not give consent for the same.

Conservative medical management can be opted for SEP patients who are hemodynamically stable. Methotrexate has been the most widely accepted treatment accepted for SEP. Bodur et al. had concluded in their study that methotrexate is the best treatment if the serum β-hCG is less than 12,000 mIU/ml, the gestational sac diameter is smaller than 8 weeks, and if there is no fetal cardiac activity [16]. Another randomized control trial compared systemic single-dose methotrexate with local methotrexate and found both the treatments to be equally successful. But this trial also observed faster resolution of pregnancy in the systemic group [17]. SEP cases where there is poor vascularization due to more amount of fibrous tissue; systemic methotrexate is less effective than local administration of the agent. Other agents like potassium chloride, hyperosmolar glucose, etoposide, and crystalline trichosanthin have also been used for local injection in SEP. Medical management has the advantage of preserving the uterus but has the demerit of bleeding for a longer time, and complete resorption of the ectopic pregnancy also is prolonged. Hence, some studies have recommended a combination of medical management with the surgical aspiration of the gestational sac. In the present study, a total of 13 cases (59%) were given methotrexate either a single dose or multiple doses. Two cases from this group needed secondary treatment for retained products of conception.

Hysteroscopic removal of SEP is also one of the treatment options available, but none of the cases included in this study had treatment with hysteroscopic removal. If the SEP is growing towards the endometrial cavity, hysteroscopic evacuation with or without prior medical management is the most appropriate. The sac can be removed under direct visualization, and in case of bleeding, simultaneous coagulation of the bleeding vessels can be done. To prevent heavy bleeding following dilatation and evacuation or hysteroscopic evacuation, hemostatic measures like balloon tamponade, vasopressin injection, uterine artery embolization, and bilateral uterine artery ligation can also be done. The limitation of treatment modalities at our institute is the nonavailability of these modern techniques for the treatment of SEP.

Laparotomy with wedge resection is preferred in hemodynamically unstable cases of SEP when there is a uterine rupture or an impending rupture. This method is considered the best by some obstetricians due to the complete removal of the SEP under direct vision. In our study, 8 cases (36%) were treated by wedge resection of the SEP. The chance of recurrence of SEP is also minimized by this method as the entire old scar, and microtubular defects are repaired. The laparoscopic approach is most suitable when the SEP is deeply implanted and is growing towards the abdominal cavity or bladder [18].

Regardless of the choice of treatment, patients with SEP require close follow-up with clinical assessment and serial measurement of serum β-hCG, ultrasonographic examinations to prevent complications, and the start of additional secondary lines of treatment.

Prevention is always better than cure. But due to the lack of sufficient data on actions for the prevention of SEP, no definite steps can be advocated. There are debates about single layer versus double-layer closure of cesarean scar to prevent scar ectopic pregnancy. Proper closure techniques may be adopted for the prevention of SEP [11].

## Conclusions

This retrospective case series has proved the role of early and accurate diagnosis of SEP for initiating the treatment in order to minimize maternal morbidity and mortality related to this rare and unusual form of ectopic pregnancy. The treatment should be individualized keeping into consideration the type of SEP, radiological image, gestational age, clinical presentation, hemodynamic status, serum β-hCG, desire for future fertility, and patient’s compliance towards follow-up. Further studies are required to rationalize the best treatment options and to understand this uncommon obstetric scenario.
